# Physicochemical properties of three Yunnan cigar tobacco leaves and identification of aroma compounds in tobacco smoke

**DOI:** 10.3389/fchem.2025.1664073

**Published:** 2025-09-08

**Authors:** Dan Chen, Haitao Chen, Haowei Sun, Yifei Man, Jie Long, Mengying Liu, Jieyun Cai, Dan Li, Shibin Xu, Kai Liu, Shuqi Wang

**Affiliations:** 1 Yunnan Tobacco Quality Inspection and Supervision Station, Kunming, Yunnan, China; 2 Beijing Key Laboratory of Flavor Chemistry, Beijing Technology and Business University, Beijing, China; 3 Chuxiong Prefecture Company of Yunan Tobacco Company, Chuxiong, Yunnan, China

**Keywords:** Yunnan cigar tobacco leaves, tobacco smoke, key aroma components, conventional chemical components, source analysis

## Abstract

**Introduction:**

This study focused on three kinds of cigar tobacco leaves (Yunxue 6, Yunxue 36, Yunxue 2) produced in Yunnan region, and through the systematic analysis of their physicochemical properties and aroma components of the smoke, the transformation pattern of aroma compounds and their origins during combustion were deeply investigated.

**Methods:**

In the experiments, gas chromatography-mass spectrometry (GC-MS) and gas chromatography-olfactometry-mass spectrometry (GC-O-MS) techniques were used to comprehensively qualitatively and quantitatively analyze the volatile constituents in the tobacco and its smoke, and combined with the conventional chemical composition detection, to reveal the physicochemical properties of Yunnan cigar tobacco and its influence mechanism on the smoke flavor.

**Results:**

Yunnan cigar tobacco exhibits distinct chemical characteristics with low sugar (0.12%–0.16%), high nitrogen (2.84%–4.56%) and alkaloid (2.32%–5.56%) contents, along with imbalanced nitrogen-alkali and potassium-chlorine ratios, affecting its combustion and sensory properties. Smoke analysis identified 144 volatile compounds, predominantly heterocyclic, aromatic and olefinic substances, with elevated pyrazines and pyridines contributing roasted, nutty and smoky notes. GC-O-MS analysis revealed 51 key aroma-active components, demonstrating greater complexity in core tobacco (YXYY) than wrapper tobacco (DHYY, PEYY).

**Discussion:**

Combustion transforms precursors (carotenoids, cedranes, phenylalanine) into characteristic aromas through pyrolysis and Maillard reactions, enhancing flavor complexity. This study first elucidates the chemical basis of Yunnan cigar’s characteristic aroma, providing theoretical support for quality improvement, process optimization and product differentiation in domestic cigar production. It fills research gaps in Chinese cigar aerochemistry and establishes a foundation for precision cultivation (e.g., chlorine regulation) and targeted fermentation processes to enhance cigar quality.

## 1 Introduction

Tobacco (*Nicotiana tabacum L.*) is a cigar product that is different from ordinary cigarettes and has a unique flavor and cultural characteristics, generally refers to the fresh tobacco leaves after drying, fermentation, aging and a series of treatments, and then manually rolled for smoking cigarettes (Fan et al., 2023), It is divided into three layers from inside to outside, namely the filler, wrapper and wrapper. In recent years, China’s economic level has been rising, the middle and high income groups have further expanded, the domestic cigar sales market is developing strongly, and the sales of domestic medium and high-end handmade cigars in 2021 exceeded 22 million, realizing 100% growth year-on-year. Cigar cigar market potential is getting bigger and bigger, and the growth momentum is strong. Cigars are loved by domestic and foreign consumers because of their unique taste, strong strength, thick and rich aroma, sweet and bitter taste and high tar content, and strong sense of satisfaction (Deng, 2021). At present, there are Sichuan, Hubei, Anhui, Hainan, Yunnan, Guangxi, Guizhou, Henan and other places planting cigar tobacco, of which Yunnan Province is located at about 23^o^N, with similar latitude and similar climatic conditions as compared with Cuba, the world’s high-quality cigar tobacco origin, abundant rainfall, abundant sunshine, good temperature and humidity harmonization, and abundant and fertile red loam and sandy loam, which has great potentials for the development of international high-quality cigar tobacco, and is an unrivaled golden region. It is a golden region with great potential for developing international high-quality cigar tobacco (Tie et al., 2024).

Due to the different sources of raw materials and production processes, cigar products have more obvious regional characteristics, aroma composition is a key factor in determining the stylistic characteristics of tobacco products, and is closely related to product quality (Zhu et al., 2024). Therefore, it is crucial to analyze the differences of cigar products from different origins in Yunnan from the perspective of aroma-causing components, so as to objectively evaluate the quality of cigar products at home and abroad, and to clarify the stylistic characteristics of cigars from different origins. In addition, the chemical composition of tobacco is very complex, and it is difficult to fully reflect the advantages and disadvantages of tobacco and tobacco products only by using certain chemical components in the non-combustible state as the index for evaluating the quality of tobacco. Therefore, the tobacco is rolled into a single cigar, through smoking combustion through pyrolysis, polymerization and a series of physical and chemical changes to form a complex smoke, to bring sensory stimulation and pleasure to the user (Mitsui et al., 2015), Cigar smoke flavor component composition and its role than the tobacco can more directly reflect the final sensory quality of cigars and style characteristics. The core physicochemical components of tobacco, such as water-soluble reducing sugars, total phytochemicals, total nitrogen, potassium ions and chloride ions, significantly influence both the sensory and intrinsic quality of tobacco. Therefore, precise measurement of these physicochemical properties in cigar tobacco is fundamentally important for subsequent investigations into smoke flavor characteristics.

Differences in combustion characteristics of different tobacco types (e.g., roasted tobacco, cigar tobacco) lead to significant differences in the composition of the smoke, and the study of their volatile compounds can provide a scientific basis for process optimization and product differentiation (Cancelada et al., 2019; Gasparyan et al., 2018), the first step in flavor analysis is the extraction and separation of flavor substances from complex food systems. Solvent-assisted flavor evaporation (SAFE)is a new flavor compounds separation technology, which is mainly composed of a distillation system and a vacuum pump system, using the rapid vaporization of solvents under low temperature and high vacuum conditions, with good qualitative and quantitative results for trace volatiles in complex foods. This technology has less damage to volatile flavor substances, less loss of heat-sensitive flavor components, and its extracted flavor is closer to the real samples, which is a more suitable extraction method for flavor substances. Sheng et al. (2023) used SPME combined with SAFE technique to extract volatile constituents from prickly pear tree, a total of 143 volatile constituents were identified by GC-MS, of which alcohols and esters were considered to be the major volatile compounds, and 45 odorant active constituents were identified by GC-O. Gas chromatography-olfactometry-mass spectrometry (GC-O-MS) consists of two powerful working units, GC-O and GC-MS, combining the features of both devices into one integrated instrument. Currently, GC-MS technology is mostly used in odor research, but GC-MS can only determine the composition and content of food aroma components, but not the key compounds that produce the aroma, whereas GC-O technology is very effective in identifying the characteristic aroma compounds as well as their aroma intensities and effects (Song and Liu, 2018). GC-O technology incorporates human sensory evaluation on the basis of precision instrumental analysis. Combined with GC-MS technology, it can more accurately and systematically determine the contribution of volatile components to the overall flavor of food products, and has been applied to a variety of fields such as food, tobacco, flavors and fragrances, as well as environmental monitoring. Jiang et al. (2023) analyzed and evaluated aged peels aged for 6 years by GC-O-MS, and a total of 42 aromatic compounds were detected, among which the aroma intensities of pungent, woody, and herbaceous compounds remained unchanged, whereas the aroma intensities of citrus and fruit compounds were gradually reduced or even disappeared.

In this study, we focused on the smoke generated from the combustion of single-filler cigarillos, and conducted an in-depth study of their aroma active components based on the molecular sensory science approach. At the same time, the types and contents of aroma compounds in tobacco and smoke were compared, aiming to comprehensively analyze the transformation pathways of compounds in the tobacco combustion process. Cigar tobacco as a kind of all-leaf hand-rolled cigarette, the quality of tobacco raw materials often affects the aroma style of cigar tobacco in smoking, therefore, the conventional chemical composition of cigar tobacco leaves was also tested to analyze the content characteristics of the conventional chemical composition of cigar tobacco leaves in Yunnan. Through these comprehensive analyses, in order to be able to assess the flavor and quality of the smoke more completely, and to provide a theoretical basis for the planting and processing of high-quality cigar tobacco leaves in China, the optimization of the process and the improvement of quality.

## 2 Materials and methods

### 2.1 Materials

**FIGURE 1:**
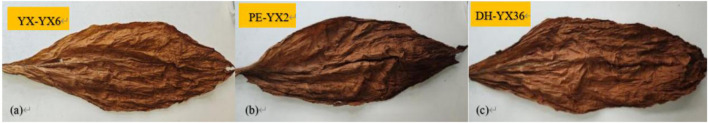
Comparison of the appearance of the three types of cigar tobacco **(a)** Yunxue No. 6 Filler Tobacco Leaves from Xinping, Yuxi; **(b)** Yunxue No. 2 wrapper tobacco leaves from Jiangcheng, Pu’er; **(c)** Yunxue No. 36 wrapper tobacco leaves from Mangshi, Dehong).

Cigar tobacco samples were provided by Yunnan Provincial Tobacco Quality Supervision and Inspection Station, selection of central eggplant core tobacco leaves of Yunxue No.6 in Yuxi City, Yunnan Province, China (YX-YX6), Pu’er Yunxue No.2 central eggplant coated tobacco (PE-YX2), Dehong Mangshi Yunxue No.36 central eggplant coated tobacco (DH-YX36). The appearance pictures of the three varieties are shown in Figure 1. In addition, the samples were subjected to the same conditions of grade, cultivation, drying and fermentation techniques. All samples were equilibrated in a constant temperature and humidity chamber (temperature 20 °C, humidity 70%–75%) for 48 h and kept for reserve.

### 2.2 Chemical reagent

Cellulose powder (particle size ≤25 µm)、2-methyl-3-heptanone (98% purity), 2-octanol (99% purity), 3-acetylpyridine (99% purity), acetophenone (99% purity), new phytodiene (90% purity) were purchased from Aladdin, the liquid nitrogen, high purity helium (purity ≥99.999%), and high purity nitrogen (purity ≥99.999%) used were purchased from Beijing Ruizhi Hanxing Technology Co, dichloromethane (analytical purity), anhydrous sodium sulfate (analytical purity) were purchased from Sinopharm Chemical Reagent, n-alkanes (C6 - C30) (chromatographic purity) were purchased from Supelco, phytanols (≥90% purity) were purchased from Psaitong, styrene (99.5% purity) was purchased from Innochem, diene nicotinic acid (98% purity), β-violet ketone (purity 97.89%) were purchased from TARGETMOL, dihydrokiwifruit lactone (purity 99.7%), cedarwood brain (purity 99.8%), 2,3′-bipyridine (purity 99.1%), 6-methyl-5-hepten-2-one (purity 97%), mescaline (purity 98%), and 2-ethylhexyl acetate (purity 98%) were purchased from Tammo Quality Inspection Company, 5,6-dihydro-6-pentyl-2H-pyran-2-one (purity 90%), and phytone (purity 98%) were purchased from Yuan Ye Company, and 2,3-dimethylpyrazine (purity 99.7%), acetic acid (purity 99.7%), isovaleric acid (purity 98%) were purchased from Genye Company. (90%), phytoncidone (98% purity) were purchased from Yuan Ye Company, 2,3-dimethylpyrazine (99% purity), acetic acid (99.7% purity), isovaleric acid (98% purity), N-methylpyrrolidone (99% purity), 3-methylpentanoic acid (98.5% purity), acetamide (99% purity), phenyl alcohol (98.5% purity), 2-pyrrolidone (98% purity), indole (98% purity).), indole (purity 99%), benzaldehyde (purity 98%), 2,3-butanediol (purity 99%), phenylacetic acid (purity 99%), 2,6-dimethylpyrazine (purity 98%) were purchased from Bailiwick Technology Company.

### 2.3 Instruments and equipment

The experimental instruments include DF-101S collector-type constant-temperature heating magnetic stirrer (produced by Gongyi Yuhua Instrument Co., Ltd.), solvent-assisted evaporation and extraction device (produced by Beijing Xinxian Jingxing Glassware Co., Ltd.), DKB-501A super constant-temperature water bath (produced by Shanghai Senxin Experimental Instrument Co., Ltd.), and XDS5 composite turbine molecular pump (produced by Edwards, U.K.), DLSB-5-30 low-temperature cooling circulating pump (produced by Shanghai Baidian Instrumentation Co., Ltd.), Wechsler shunt column (produced by Beijing Halfsia Science and Technology Development Co., Ltd.), BF-2000 nitrogen blow-drying instrument (produced by Beijing Bafang Century Science and Technology Co., Ltd.), TRACE1310-ISQQD gas chromatography - mass spectrometry coupled with (produced by the United States of America Thermo Fisher Scientific), electronic analytical balance (produced by the United States of America), electronic analytical balance (produced by the United States of America Thermo Fisher Scientific). Ltd.), electronic analytical balance (produced by Beijing Sartorius Instrument Systems Co., Ltd.), DB-WAX capillary chromatography column (produced by Thermo Fisher Scientific, Ltd.), ODP3 sniffer (produced by Chester (Shanghai) Trading Co., Ltd.), and cigar thermostat cabinet (produced by Haier Commercial Electrical Appliances). In addition, the instruments used to roll cigars and collect smoke are cigar shaper (Secret 132), cigar cutter, cigar ligature, all purchased from Wuhou District Jinggong Advertisement Design Service Department, and cigar smoking machine with single aperture (CSM1000) purchased from Hefei Research Institute of Physical Sciences, Chinese Academy of Sciences.

### 2.4 Detection of chemical composition

Cigar leaf samples were subjected to vein removal and leaf body fine cutting, and then standardized quantitative testing was carried out for the six core physicochemical indexes (total sugars, reducing sugars, total phytobases, total nitrogen, potassium and chlorine) in accordance with the standards of the State Tobacco Industry. The total sugar and reducing sugar content is determined according to the “Determination of Water-soluble Sugar in Tobacco and Tobacco Products by Continuous Flow Method” (YC/T159-2019); the total phytochemical content is determined according to the “Determination of Total Phytochemical Alkaloid in Tobacco and Tobacco Products by Continuous Flow (Potassium Thiocyanate)” (YC/T468-2021); the total nitrogen content is determined according to the “Determination of Total Nitrogen in Tobacco and Tobacco Products by Continuous Flow Method” (YC/T468-2021). Determination of Total Nitrogen in Tobacco and Tobacco Products by Continuous Flow Method (YC/T161-2002); Potassium content shall be determined according to “Determination of Potassium in Tobacco and Tobacco Products by Continuous Flow Method” (YC/T217-2007); Chlorine content shall be determined according to “Determination of Chlorine in Tobacco and Tobacco Products by Continuous Flow Method” (YC/T162-2011). Chlorine content is determined according to Continuous Flow Method for the Determination of Chlorine in Tobacco and Tobacco Products (YC/T162-2011).

### 2.5 Flue gas collection

Rolling of single-feed cigarillos: The above three types of cigarillos were rolled into three single-feed cigarillos each: the whole cigarillo leaf was sprayed with water and wetted and processed to reach the softness and hardness that could be rolled, and then combined with a cigarillo rolling mold to roll single-feed cigarillos by hand, and then the sample cigarillos (circumference of 47 mm × length of 110 mm, 6.0 ± 0.2 g) were placed in a cool and ventilated place for 3 days after the completion of the preparation and then put into a (20 ± 2)°C, relative humidity (70 ± 2)% for 72 h. The samples were then placed in a cool and ventilated place for 3 days, and then equilibrated at (20 ± 2)°C, relative humidity (70 ± 2)% for 72 h.

Prepare the Single Orifice Smoker by placing a 55 mm Cambridge filter into the smoke trap with the rough side facing the air inlet and checking for proper assembly. Turn on the air compressor to make the air pressure ≥0.5 MPa and start the warm-up program of the smoker for 30 min carry out an air leakage test to ensure the integrity of the air path of the trap. The accuracy of the suction volume was then tested and the suction volume parameters were calibrated using the soap film displacement in the soap film flow meter.

The use of single-orifice smoking machine: three kinds of cigar smoke (YXYQ, DHYQ, PEYQ) were obtained by smoking the three kinds of single-feed cigar cigars rolled as mentioned above in turn: the smoking parameters of the cigarettes were set in accordance with the CI international standard (smoking capacity of 55 mL, smoking frequency of 30 s, duration of smoking of 2 s, and number of fixed mouths of 50 mouths). The particle-phase material in the mainstream flue gas was retained by Cambridge filter sheets, while the capture of the gas-phase material in the flue gas required the construction of a continuous capture system, with three solvent absorption bottles in series at the rear end of the trap, and 80 mL of re-distilled dichloromethane solution added respectively. A conditioned cigar stick was inserted into the aperture of the trap clamping device, ensuring that the end of the stick was in contact with the porous buffer gasket, and the detector scale was adjusted to 28 mm of the stick. The ignition program was initiated to start suctioning, and at the end of the suction, 2 puffs of blank suction were performed to remove the residual components in the orifice, and the cigarette butt was removed to end the suctioning operation.

### 2.6 Extraction of volatile substances

Three vials of extraction solution were mixed, and Cambridge filter discs were placed in the extraction solution for 1 h. 80 μL of 2-octanol solution (1.26 μg/μL) was added as an internal standard. The volatile components were separated using the SAFE method. After 10 g of cigar tobacco leaves were crushed by a pulverizer and put into a conical flask, 100 mL of redistilled dichloromethane was added as the extraction solvent and 50 μL of 2-octanol solution (1.26 μg/μL), 50 μL of 2-methyl-3-heptanone (concentration of 1 μg/μL) were added as the internal standard, which were sealed and then extracted continuously for 1 h and then extracted with a thermostatic magnetic stirrer (1000 r/min) for 1 h and 1 h of extraction with a constant temperature (1.5 μm) at room temperature. The extract was then continuously extracted for 1 h at room temperature using a constant temperature magnetic stirrer (speed 1000 r/min) and filtered under vacuum. The filtrate residue was collected and the above steps were repeated by adding 100 mL of redistilled dichloromethane, and the two extracts were combined. The extracts were introduced into a solvent-assisted distillation unit to complete the phase separation of the volatile components under ultra-high vacuum (10-5 mbar), and the obtained fractions were dehydrated by anhydrous sodium sulfate, further concentrated to 1.5 mL by Vigreux fractionation unit (50 cm × 1 cm) and filtered by microporous membrane, and then concentrated by nitrogen purge to 1 mL. The samples were sealed and stored in an ultra-low temperature refrigerator at −40 °C. The samples were stored in an ultra-low temperature refrigerator. The samples were sealed and stored in an ultra-low temperature refrigerator at −40 °C for subsequent analysis.

### 2.7 GC-O analysis

A trained sensory panel (n = 3) was employed for GC-O analysis following a standardized protocol. Panelists were selected based on olfactory acuity (n-butanol threshold test) and odor identification performance. Prior to analysis, they underwent 40 h of training, including exposure to reference compounds (e.g., 2-acetylpyrazine, β-damascenone) and smoke extracts to calibrate intensity perception (0–3 scale) and descriptor consistency. Validation confirmed intra-panelist reproducibility (>90% descriptor agreement, intensity variation < ± 0.5 units) and inter-panelist reliability (Intraclass Correlation Coefficient = 0.82, Fleiss’ Kappa = 0.75). Only odorants detected by ≥2 panelists (FD% ≥ 66.7%) were considered significant. During analysis, the GC effluent was split (1:1) between MS and sniffing port (ODP3), with panelists recording retention time, intensity, descriptor, and duration of each odor event. Detection Frequency (FD%) served as the primary metric for odor activity. The rigorous protocol ensured reproducible identification of key odor-active compounds in cigar smoke.

### 2.8 GC-O-MS analysis

A TG-WAX (30 m × 0.25 mm × 0.25 μm, 30 m) column was used to achieve the separation of volatile components, and the mass spectrometry detector was synchronously connected to the olfactory detection port via a Y-shaped splitter (split ratio 1:1). Each sample was recorded by three trained sensory evaluators for sniffing. The temperature gradient of the GC column temperature chamber was programmed as follows: initial temperature of 50 °C, constant temperature for 2 min, ramping up to 120 °C at 6 °C/min, holding for 4 min, then ramping up to 200 °C at 4 °C/min, and then ramping up to the final temperature of 240 °C at 8 °C/min, holding for 8 min. Ultra-high purity helium (99.99%) was used as the carrier gas, with a constant flow rate of 1.0 mL/min, and the injection volume of 1 µL was 1 µL. The mass spectrometry conditions were set as follows: EI ionization source, ionization energy of 70 eV, ion source temperature of 250 °C, mass range of 40–550 m/z, full scan, solvent delay of 3.5 min.

### 2.9 GC-MS analysis

A single quadrupole mass analyzer coupled with a gas chromatographic system was used to construct a platform for the detection of volatile components. The same TG-WAX capillary column was used as that used in the GC-O-MS analysis, and the temperature setting and mass spectrometry conditions were the same as those in “2.7 GC-O-MS analysis”.

### 2.10 Qualitative and quantitative analysis

Qualitative methods: qualitative analysis of aroma compounds was performed by (1) similarity analysis based on the NIST14 Library database with Xcalibur software data processing; (2) linear retention indices (RI) of aroma compounds determined on a DB-WAX column and compared with reference values (allowed deviation ±20); (3) odor description with purchased standard compounds matching; and (4) comparing the consistency of chromatographic retention behavior between the target and the standard under the same analytical conditions of GC-MS. The retention index (RI) was calculated from the retention times of a series of n-alkanes (C6-C30) in GC-MS by Eq:

$$RI=100\times \left[n+\frac{\log t^{\prime \left(i\right)}-\log t^{\prime \left(n\right)}}{\log t^{\prime \left(n+1\right)}-\log t^{\prime \left(n\right)}}\right]$$

where: t′(i) - retention time of the unknown compound; t′(n) and t′ (n+1) - retention time of an orthoalkane with n and n+1 carbon atoms, the retention time of n-alkanes.

Quantitative method: the content of the aroma active compounds was calculated by the internal standard semiquantitative method [4]: by dividing the peak area of the target compound by the peak area of the internal standard and multiplying this ratio by the concentration of the internal standard, assuming a correction factor of one.

$$c\left(i\right)=\frac{A\left(i\right)}{A\left(s\right)}\times c\left(s\right)$$

where: c(i) represents the concentration of the unknown aroma compound i; A(i) represents the peak area of the unknown aroma compound i; A(s) represents the peak area of the internal standard compound s; and c(s) represents the concentration of the internal standard compounds.

### 2.11 Statistical analysis

Three parallel experiments were performed and the results are expressed as mean ± standard deviation. The datasets of the compounds were processed and analyzed using Origin2024 and Excel software; Chemdraw 2023 software was used to make the structural formulae of the compounds.

## 3 Results and discussion

### 3.1 Chemical content results

The conventional chemical composition of Yunnan cigar tobacco leaves was statistically analyzed and the results are shown in Table 1. The results showed that the total sugar content of the central leaf of Yunnan cigar tobacco leaf ranged from 0.12% to 0.16%, reducing sugar content was not detected (below the detection limit), total phytobasic alkaloids ranged from 2.32% to 5.56%, total nitrogen content ranged from 2.84% to 4.56%, chlorine ranged from 1.16% to 1.80%, potassium ranged from 4.12% to 4.86%, and total phytobasic alkaloids, total nitrogen content varied more and the rest varied less.

**TABLE 1:** Conventional chemical content of three cigar tobacco types.

Sample	Moisture (%)	Total nitrogen (%)	Water-soluble reducing sugar (%)	Total water-soluble sugars (%)	Total phytanine (%)	Chlorine (%)	Potassium (%)
DHYY	7.76	4.19	-	0.12	3.38	1.80	4.43
PEYY	8.18	4.56	-	-	5.56	1.16	4.12
YXYY	7.60	2.84	-	0.16	2.32	1.49	4.86

Note: “-”indicates not detected, the same as below.

#### 3.1.1 Sugar

Sugar, a product of photosynthesis, is an important intrinsic indicator of tobacco quality. To be precise, sugar can mask undesired odors by producing acids, which can neutralize or attenuate the pungency of the smoke (Yan et al., 2022). In addition, sugar has a positive impact on the quality of aroma, which can be converted into flavor components (e.g. furfural, furans) through the Melad and caramelization reactions (Banožić et al., 2020; Li T. X. et al., 2022), gives the cigar caramelized and roasted aromas and enhances the sweetness of the aftertaste. Li et al. (2016) believed that total sugar content was significantly correlated with positive sweet flavor, and reducing sugar was significantly positively correlated with caramelized sweet flavor. Generally, the total sugar content of high-quality roasted tobacco is 18%–22%, and the reducing sugar content is 16%–18% (Jin et al., 2021),about 10 times more than cigar tobacco, with a much higher sugar content. The reason for the low sugar content of cigar tobacco may be that there is no supply of exogenous energy substances to the tobacco during the drying period, and the leaf begins to consume a large amount of its own sugar to provide energy, degraded to monosaccharides and aroma precursors (Yi et al., 2023). As shown in Table 1., the total sugars detected in DHYY and YXYY were 0.12% and 0.16%, respectively, which were both relatively low, while no total sugars were detected in PEYY (limit of quantification: 0.021%). Reducing sugars were not detected in any of the three samples, thus concluding that the sugar content of Yunnan cigar tobacco is generally low.

#### 3.1.2 Total nitrogen and total phytochemicals

**FIGURE 2:**
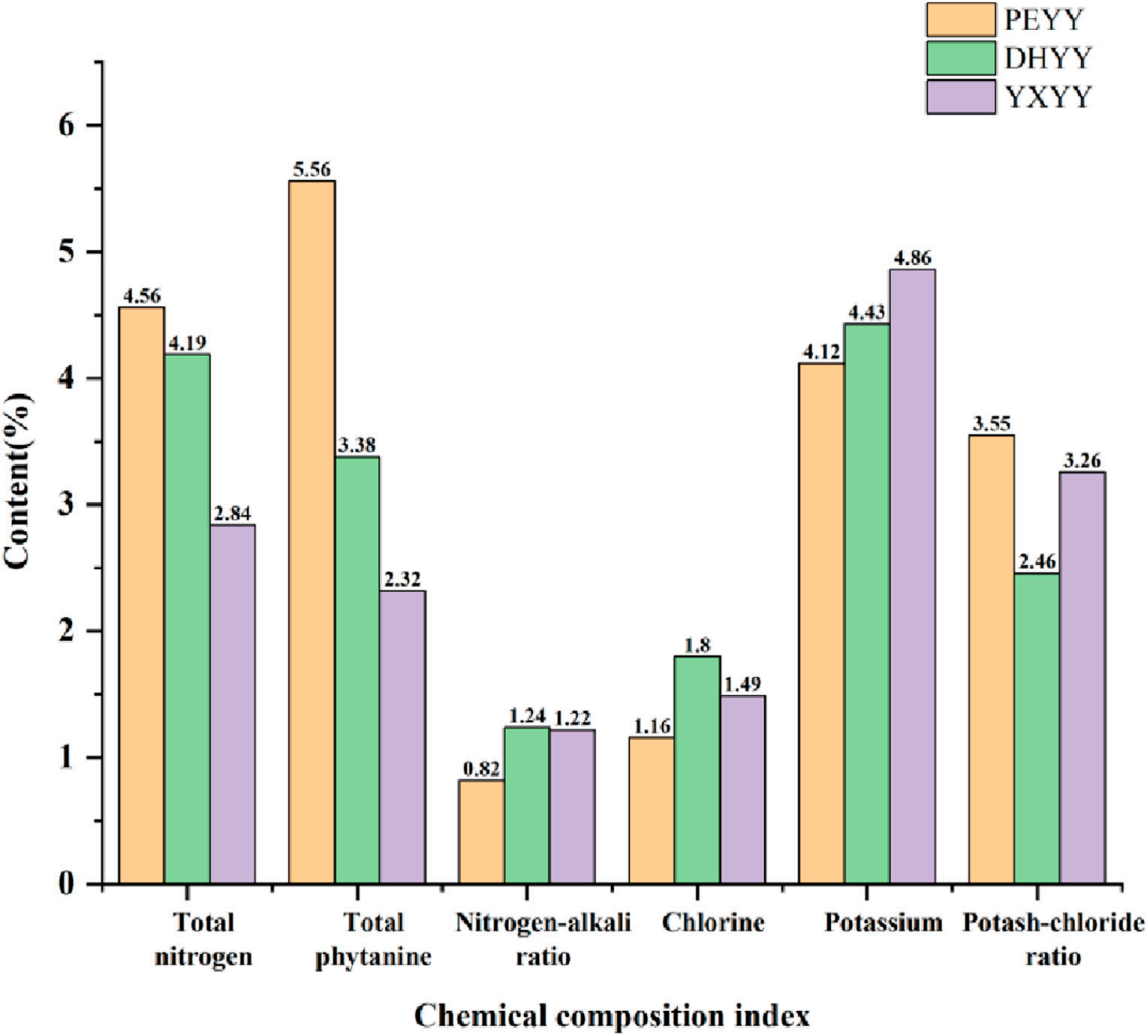
Total nitrogen, total phytochemicals, chlor-alkali ratio, chlorine, potassium, potash-chloride ratio in cigar tobacco samples.

The total amount of nitrogen in cigar tobacco characterizes the integrated level of its nitrogenous metabolites, covering protein macromolecules, amino acid precursors, volatile alkaloids and chlorophyll and other components. These nitrogen compounds (e.g. pyridine, amide, etc.) undergo pyrolytic transformation during combustion, generating alkaline pyrolysis products with a high pH value, which significantly enhance the sensory irritation of the smoke and form a burnt and spicy character, and play a key role in regulating the layering and richness of the aroma of the smoke (Yi et al., 2023). Compared with other types of tobacco products, cigarillos have a relatively high nitrogen content (Hu et al., 2023),which is more stimulating, and cigars are more suitable for consumers seeking strong physiological sensations due to their high nitrogen characteristics. When the total nitrogen content of the tobacco is 1.6%–2.2%, the aroma and taste are better, and when the total nitrogen content is 3.0%, the smoking quality is the best (Yang et al., 2022). Compared with high-quality cigar tobacco leaves, DHYY and PEYY had high total nitrogen contents of 4.19% and 4.56%, respectively, while YXYY had a total nitrogen content of 2.84%, which was close to the optimal value. The accumulation of total nitrogen was closely related to fertilization. Studies have shown (Guan et al., 2023), hat excessive nitrogen application significantly increases total nitrogen content, but through the dosing of trace elements (e.g., magnesium and sulfur), carbon and nitrogen metabolism can be optimized to reduce total nitrogen and enhance sugar content, improving the flavor of tobacco. Nicotine is the main component of total phytochemicals, which directly determines the physiological strength and vigor of the smoke. If the nicotine content in the smoke is too low, the strength is small and the smoking flavor is bland, and if the content is too high, the strength is large and the stimulant is enhanced, resulting in a pungent flavor (Yang et al., 2024). Cigar tobacco leaves generally have a high nicotine content, which is suitable for consumers seeking intense satisfaction, but excessive amounts can cause a burning sensation in the mouth. High-quality cigar tobacco generally has a nicotine content of around 1%-2% (Yang et al., 2022),the total phytochemical content of YXYY was 2.32%, while DHYY (3.38%) and PEYY (5.56%) had high total phytochemical content, which may have led to higher irritation during vaping. Nitrogen to alkali ratio is a key parameter in the evaluation of the quality of cigar tobacco, which is higher or lower than the appropriate value will lead to poor aroma quality. If it is too high, the aroma is poor, the strength is weak and the aftertaste is not pleasant; if it is too low, the aroma is insufficient, the smoke is not full, and the dough formation is poor (Kai et al., 2020), nitrogen to alkali ratio between 3-4 is optimal. From Figure 2 it can be seen that the nitrogen-base ratios of the three samples were all under 2%, with PEYY being the lowest, indicating that the range of nitrogen-base ratios of Yunnan cigar tobacco leaves was low compared with that of high-quality cigar tobacco leaves, which is consistent with the conclusion of Liu et al. (2022). In summary, Yunnan cigar tobacco leaves were characterized by high total nitrogen content, high total phytochemicals, and low nitrogen-alkali ratios, with a dysfunctional nitrogen-alkali ratio.

#### 3.1.3 Potassium to nitrogen ratio

As a typical chlorine-sensitive crop, cigar tobacco has a significant physiological response to chloride accumulation. When the chlorine content of tobacco is too high, the combustibility is significantly reduced, resulting in loose ash, easy to extinguish, heavy off-gassing, and low utilization value. However, a moderate amount of chlorine (0.4%–0.6%) enhances the elasticity and moisturizing properties of the tobacco (Wang et al., 2022), its mass fraction of 1% or less is appropriate, chlorine more than 1% will burn poorly, there will be black ash extinguishing phenomenon, quality deterioration (Tiecher et al., 2023). The chlorine content of the three types of tobacco exceeded 1%, and the chlorine content of DHYY was the highest at 1.8%, which indicated that there was a general phenomenon of chlorine accumulation in Yunnan cigar tobacco, which could easily lead to poor combustion and black ash extinguishing phenomenon, and severely restricted its combustion and smoke quality stability. Potassium is the main component of tobacco ash and an important quality element of cigar tobacco, which not only improves the combustibility, flavor and oil content, but also reduces the tar content, which plays an important role in improving the availability of tobacco (Gao et al., 2024). The presence of potassium favors the complete combustion of tobacco, while the presence of chlorine has a tendency to slow down combustion, making potash-to-chlorine ratio an important indicator of cigar combustibility (Yin et al., 2018). High potassium and low chlorine (>4) are the characteristics of high quality (Wang, 2003), and tobacco with a potassium to chlorine ratio below 2 is easy to flame out. The potassium-to-chlorine ratio of roasted tobacco is generally higher than that of cigars. Xia Yuzhen et al. found that the potassium-to-chlorine ratio of roasted tobacco in Yunnan and Fujian is 20% higher than that of cigars (Xia et al., 2015). From Figure 2 it can be seen that the potassium-chlorine ratios of DHYY, PEYY and YXYY were 2.46, 3.55 and 3.26, respectively, which were above 2 but did not exceed 4, indicating that the combustibility of the three kinds of tobacco did not reach the standard of high-quality tobacco. In summary, Yunnan cigar tobacco is characterized by high chlorine content, low potassium-chlorine ratio and disordered potassium-chlorine ratio.

### 3.2 GC-MS analysis results

**FIGURE 3:**
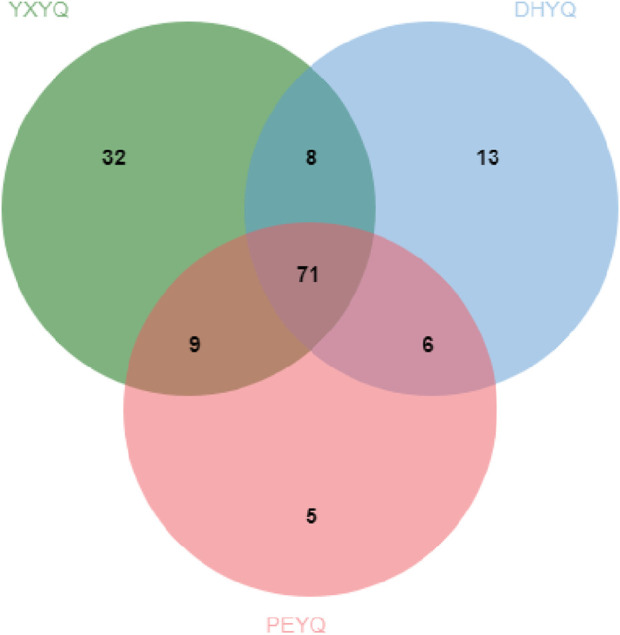
Wayne diagrams for volatile compounds in three flue gases.

Processing and analyzing the GC-MS data revealed 144 volatiles, including heterocyclics (46), aromatics (37), olefins (18), ketones (19), acids (7), alcohols (7), amides (3), and nitriles (7), a total of eight major categories, in which heterocyclics, aromatics, and olefins played an The quantitative relationships of the volatile components identified in the three samples are shown in Figure 3. A total of 71 components were found in the different cigar smoke samples, indicating that the volatiles of the smoke samples did not differ much in composition. The types of compounds identified in the concentrated extracts of different smoke gases varied, among which 120, 98 and 91 volatiles were identified from YXYQ, DHYQ and PEYQ, respectively, with volatile contents of 5131.226, 3045.91, and 2976.645 μg/L. It can be seen that the types and contents of volatile compounds in YXYQ were much higher than those in DHYQ and PEYQ, probably due to the fact that YXYQ is obtained by rolling and smoking cigar filler tobacco, while DHYQ and PEYQ are obtained by rolling and smoking wrapper tobacco, and the filler determines the overall level and stylistic characteristics of a cigar, and the aroma is richer than that of wrapper tobacco.

**FIGURE 4:**
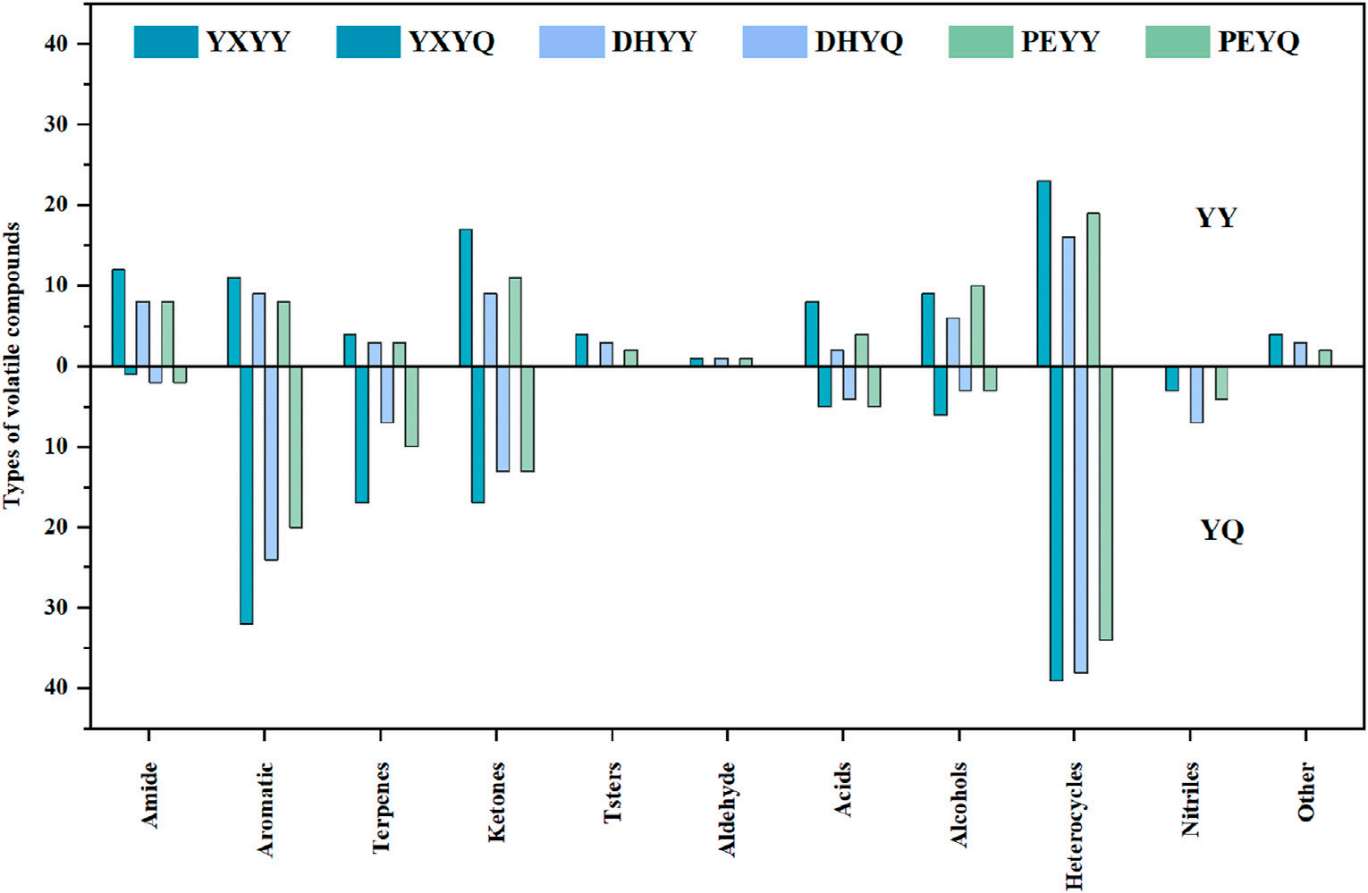
Comparison of tobacco and flue gas volatile compound species.

Compared to tobacco, the volatile compounds in smoke increased significantly (Figure 4.), with an increase in heterocyclic, olefinic, and aromatic compounds and a decrease in amides, esters, and alcohols, as well as an increase in nitrile compounds in the smoke. Among the volatile components in cigar tobacco and smoke, heterocyclic compounds showed significant advantages in terms of species diversity and content. These compounds as the key aroma substances in the formation of the characteristic flavor of tobacco, such as pyrazines have roasted, nutty and burnt aroma, which can increase the roasted aroma of the smoke, and is an important source of “mellow” in high-end cigars, and the content of pyrazine compounds in the smoke is much higher than that in the leaf, which is probably produced by pyrolysis of proteins, sugars and other compounds in the tobacco leaf during combustion (Scalone et al., 2019). The pyrazine compounds in cigarette smoke are much higher than those in tobacco leaves, and may be produced by pyrolysis of proteins and sugars in tobacco leaves during combustion (Scalone et al., 2019), while pyrazine compounds in tobacco leaves are produced by microbial degradation of nitrogen-containing compounds in the process of fermentation and aging (Koehler et al., 1971). Pyrazines are important nitrogen-containing heterocyclic compounds in tobacco and its smoke, and their aroma profile is dominated by pungent, smoky, and woody aromas, which increase the smoky aroma of the smoke and enhance the sense of aging (Gancarz et al., 2022). Their types and contents in the smoke are also much higher than those in the tobacco, which may be due to pyridine compounds generated by pyrolysis of proteins and cellulose in the tobacco during combustion, and also due to the meladic reaction of amino acids (e.g., aspartic acid, glutamic acid) and reducing sugars in the tobacco during combustion, which generates alkyl pyridine compounds (El-Hage et al., 2018). Aromatic compounds, as an important aroma active component of tobacco flavor composition, were detected in tobacco metabolites and pyrolysis components of smoke in a greater variety and content, such as phenethyl alcohol with rose-like floral characteristics, which can effectively enhance the sweet, clear and floral rhythms of cigarettes, and achieve a synergistic enhancement of aromatic complexity and comfort; phenol with the iconic smoky flavor of cigars; and benzaldehyde with the sweet aroma of almonds, which can enhance the Cigar smoke sweet aroma complexity, there are 2 main generation pathways: one is during combustion, lignin side chain breakage to generate monocyclic phenols, the second is the reaction between sugars and amino acids, including the Melad reaction, glucose and phenylalanine reaction to generate benzaldehyde (almond aroma), and the Strecker degradation (phenylalanine to generate phenylglycolaldehyde) (Li X. et al., 2022). In addition, more types and contents of aroma compounds in tobacco and smoke are olefins, and the types and contents of olefinic compounds increased after the tobacco was burned, monoterpenes such as limonene, lauricene contributed floral and fresh fruity aroma to the smoke, and sesquiterpenes such as α-farnesene had grassy aroma, which enhanced the freshness and fruity level of the smoke. One part is produced by lipid oxidation and pyrolysis: lipids in tobacco (e.g. linoleic acid, linolenic acid) undergo oxidative fracture during combustion to produce small molecule olefins (McGrath et al., 2007). A portion is obtained by thermal conversion of terpenes. The decrease in amides in the flue gas may be due to the increase in nitriles in the flue gas as a result of the deamination reaction that occurs during the combustion of cigar tobacco to release NH_3_, and the dehydration reaction to produce nitriles. The content of esters and alcohols decreased significantly during the combustion process and were mainly converted into small molecular compounds such as acids, aldehydes, and olefins.

### 3.3 GC-O-MS analysis results

**FIGURE 5:**
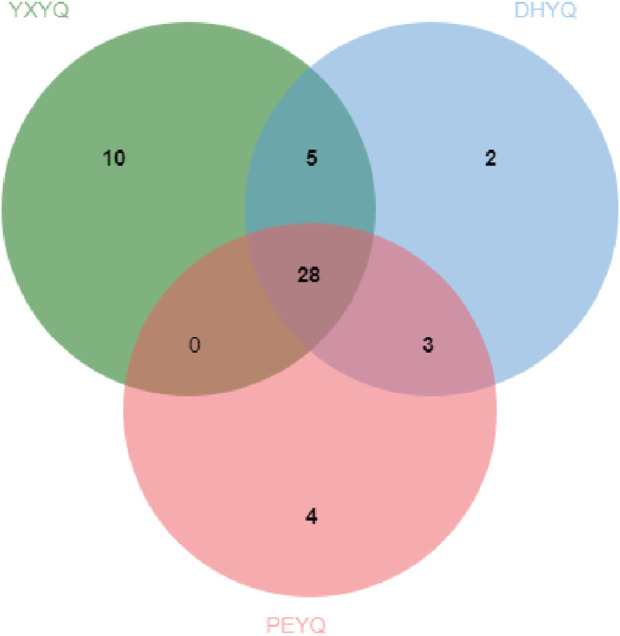
Wayne diagrams of aroma compounds in three flue gases.

A total of 51 aroma compounds were sniffed and characterized by GC-O-MS for the three flue gases (see Figure 3.), of which 43 aroma compounds were sniffed in YXYQ, 38 aroma compounds were sniffed in DHYQ, and 35 aroma compounds were sniffed in PEYQ. A total of 28 aroma compounds were the same among the three types of smoke (Figure 5.), indicating that the aroma substances of the smoke samples did not differ much in composition.

**FIGURE 6:**
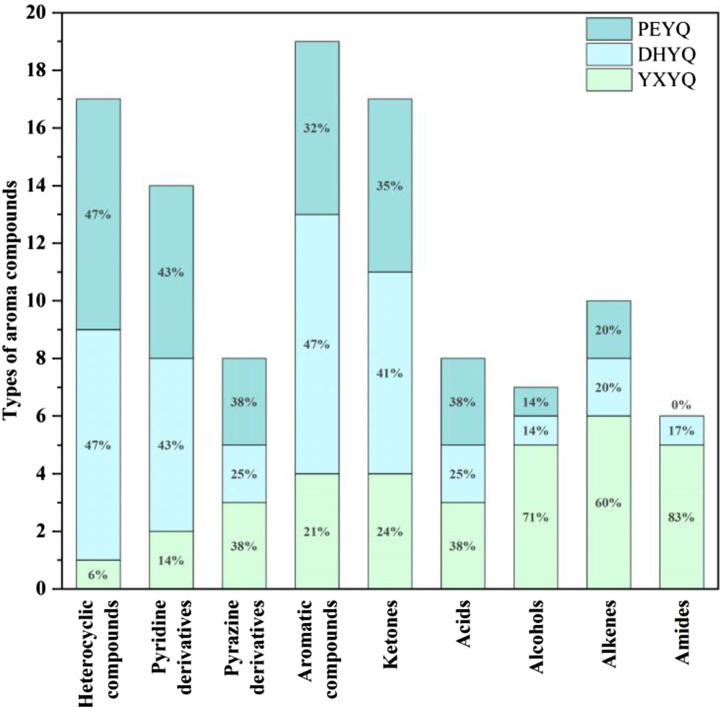
Types of aroma compounds in three types of flue gas.

The aroma compounds sniffed in YXYQ consisted of 17 heterocyclic compounds (Figure 6.), including 7 pyridines and 2 pyrazines, 10 aromatic compounds, 10 ketones, 2 acids, and 4 terpenoids. Heterocyclic compounds were the most abundant, in which pyridines showed coal tar aroma with an unpleasant odor, which is the main source of tobacco aroma, pyrazines mostly showed nutty and roasted aroma, followed by ketones and aromatic compounds, ketones mostly showed green and fruity aroma, and aromatic compounds mostly showed floral, fruity and other aromatic aroma. Among the aroma compounds that appear only in YXYQ are lauricene (woody, pine), 2,6-dimethyl-2,4,6-octatriene (sweet aroma), acetic acid (sour aroma), 3,4-dimethyl-2-cyclopentenone (sweet aroma, fruity aroma), 2-acetylfuran (cocoa aroma), 3,4,4-trimethylcyclopent-2-enone (pine, woody aroma), 3-ethyl-2-cyclopenten-1-one (sweet aroma), indoline (floral aroma), 3-acetylpyridine (sweet, fruity aroma), and phenethyl alcohol (floral aroma). The organoleptic properties of these aroma components were very rich, suggesting that YXYQ is complex in flavor and has a unique flavor profile.The aroma compounds detected by olfaction in DHYQ included 16 heterocyclic compounds, 9 aromatic compounds, 7 ketones, 1 alcohol, 2 acids, 2 terpenoids, and 1 amide. The overall aroma of DHYQ was weaker compared to YXYQ, and two of the aroma-active compounds that were present only in DHYQ but not in the other samples were acetamide, which has a musty flavor, and 1-(2-furanylmethyl)-1H-pyrrole, which has a tobacco flavor and burnt aroma. These may be some of the important compounds in DHYQ that have fermented and smoky aroma.The aroma compounds sniffed in PEYQ included 17 heterocyclic compounds, 6 aromatic compounds, 6 ketones, 1 alcohol, 3 acids, and 2 terpenes. The overall variety of compounds in PEYQ was less than the first two types of fumes, which were mainly ketones, heterocyclic compounds, and aromatic compounds. Four aroma compounds not detected in the other samples were sniffed in the PEYQ sample, namely 3-ethyl-2,5-methylpyrazine with a saucy aroma, propionic acid with a sour aroma, 5-ethyl-2-furaldehyde with a malty aroma, and 4-isopropylphenol with a pungent, woodsy aroma.

### 3.4 Quantitative analysis results

**FIGURE 7:**
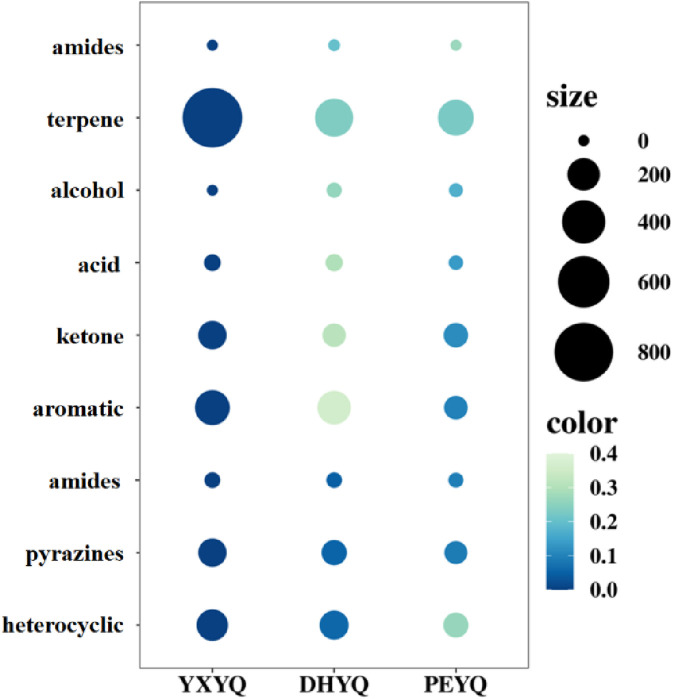
Comparison of the content of aroma compounds in three types of flue gas.

In order to determine the content of the aroma active ingredients in the three samples, the quantitative analysis was carried out by GC-MS-O using 2-octanol as an internal standard, and the results are shown in Table 2. In order to visualize the content of each type of aroma active ingredient in the flue gas, the content bubble diagram was plotted, and the results were shown in Figure 7. The more compounds were present, the larger the bubbles were. The quantitative results showed that the contents of 28 kinds of aroma active ingredients in different flue gases accounted for 86.5%–93.79%, which made them have similar aroma characteristics; however, there were differences in the contents of some aroma compounds, which made the intensity of their aroma characteristics different.

**TABLE 2:** Three types of cigar smoke aroma substances and their contents.

No.	CAS	Compound	Aroma	Identifcation methods	Content (μg/L)		
					YXYQ	DHYQ	PEYQ
1	98-01-1	Furfural	Graininess	MS/RI/O	3.175 ± 0.05	0.474 ± 0.142	0.238 ± 0.046
2	1192-62-7	2-Acetylfuran	Cocoa	MS/RI/O	7.087 ± 0.274	3.155 ± 0.545	2.857 ± 0.069
3	109-97-7	Pyrrole	Nuts	MS/RI/O	35.841 ± 0.787	19.747 ± 1.392	21.482 ± 0.881
4	23074-10-4	5-Ethyl-2-furaldehyde	Malt fragrance	MS/RI/O	—	—	4.443 ± 0.477
5	496-15-1	Indoline	Floral	MS/RI/O	2.932 ± 1.178	—	—
6	120-72-9	Indole	Fecal odor	MS/RI/O	46.035 ± 4.657	55.066 ± 3.313	28.382 ± 1.159
7	581-50-0	2,3′-Bipyridine	Tobacco, caramelized	MS/RI/O	22.846 ± 2.344	33.909 ± 2.201	19.142 ± 1.102
8	1438-94-4	1-Furylpyrrole	Tobacco, caramelized	MS/RI/O	—	0.728 ± 0.083	—
9	532-12-7	Myosmine	Tobacco, caramelized	MS/RI/O	44.867 ± 2.167	24.114 ± 3.059	15.534 ± 0.517
10	487-19-4	β-Nicotyrine	Tobacco, caramelized	MS/RI/O	21.659 ± 2.676	11.951 ± 0.71	8.755 ± 0.307
11	109-06-8	2-Picoline	Tobacco	MS/RI/O	7.551 ± 0.504	0.642 ± 0.663	7.859 ± 0.994
12	100-71-0	2-Ethylpyridine	Fishy	MS/RI/O	21.595 ± 1.644	3.762 ± 0.273	4.643 ± 0.533
13	108-99-6	3-Picoline	Raw peanut	MS/RI/O	58.068 ± 0.861	68.281 ± 0.256	31.866 ± 2.451
14	589-93-5	2,5-Dimethylpyridine	Sweet, rice	MS/RI/O	14.744 ± 0.218	7.379 ± 0.038	7.648 ± 0.465
15	108-47-4	2,4-Lutidine	Fumigate	MS/RI/O	13.976 ± 1.333	9.507 ± 0.245	12.02 ± 0
16	536-78-7	3-Ethylpyridine	Tobacco, leather	MS/RI/O	22.111 ± 0.226	12.946 ± 1.871	14.431 ± 1
17	350-03-8	3-Acetylpyridine	Sweet, fruity	MS/RI/O	2.253 ± 0.156	—	—
18	109-08-0	2-Methylpyrazine	Roasty	MS/RI/O	14.527 ± 1.94	15.322 ± 3.639	9.781 ± 0.574
19	14667-55-1	Trimethyl-pyrazine	Roasty	MS/RI/O	6.859 ± 0.289	5.168 ± 0.087	5.604 ± 0.553
20	13360-65-1	3-Ethyl-2,5-dimethylpyrazine	Sauce	MS/RI/O	—	—	1.612 ± 0
21	100-41-4	Ethylbenzene	Aromatic	MS/RI/O	26.44 ± 4.698	33.079 ± 0.834	—
22	100-42-5	Styrene	Floral	MS/RI/O	32.596 ± 6.176	35.157 ± 1.183	48.005 ± 4.224
23	98-86-2	Acetophenone	Pungent	MS/RI/O	18.86 ± 1.571	7.894 ± 0.589	8.259 ± 0.849
24	122-00-9	4′-Methylacetophenone	Sweet	MS/RI/O	5.644 ± 0.574	1.572 ± 0.403	—
25	60-12-8	Phenethyl alcohol	Floral	MS/RI/O	18.378 ± 0.309	—	—
26	95-48-7	o-Cresol	Tobacco, caramelized	MS/RI/O	82.927 ± 0.449	89.41 ± 2.857	—
27	99-89-8	4-Isopropylphenol	Pungent, wood	MS/RI/O	—	—	8.573 ± 0.594
28	620-17-7	3-Ethylphenol	Tobacco, caramelized	MS/RI/O	31.225 ± 0.57	28.034 ± 0.869	15.652 ± 1.245
29	91-20-3	Naphthalene	Acrimony, choke	MS/RI/O	11.306 ± 0.84	15.118 ± 1.302	—
30	90-12-0	1-Methylnaphthalene	Herbal, cool	MS/RI/O	2.965 ± 2.71	1.166 ± 0.044	0.338 ± 0.442
31	91-57-6	2-Methylnaphthalene	Sweet, floral	MS/RI/O	7.389 ± 0.085	3.659 ± 0.232	3.954 ± 0.454
32	625-33-2	3-Penten-2-one	Sweet	MS/RI/O	18.311 ± 1.429	10.746 ± 1.676	54.191 ± 1.037
33	592-20-1	Acetoxyacetone	Nutty	MS/RI/O	29.061 ± 4.049	11.822 ± 1.164	11.403 ± 0.385
34	30434-65-2	3,4,4-Trimethylcyclopent-2-enone	Pines, wood	MS/RI/O	3.433 ± 0.027	—	—
35	30434-64-1	3,4-Dimethylcyclopent-2-en-1-one	Sweet, fruity	MS/RI/O	5.57 ± 0.533	—	—
36	2758-18-1	3-Methylcyclopent-2-en-1-one	Sweet	MS/RI/O	22.103 ± 0.056	31.305 ± 0.535	9.822 ± 0.635
37	5682-69-9	3-Ethylcyclopent-2-en-1-one	Sweet	MS/RI/O	4.573 ± 0.641	—	—
38	54868-48-3	(E)-5-Isopropyl-8-methylnona-6,8-dien-2-one	Tea, green	MS/RI/O	36.566 ± 6.664	12.02 ± 1.055	12.392 ± 1.603
39	80-71-7	Methyl cyclopentenolone	Sweet, caramelize	MS/RI/O	0.142 ± 0	6.471 ± 0.05	—
40	21835-01-8	3-Ethyl-2-hydroxy-2-cyclopenten-1-one	Sweet, caramelize	MS/RI/O	10.631 ± 1.691	3.978 ± 0.747	1.181 ± 0.256
41	502-69-2	Fitone	Tobacco	MS/RI/O	10.665 ± 0.809	6.395 ± 0.614	4.896 ± 0.381
42	64-19-7	Acetic acid	Sour	MS/RI/O	24.09 ± 1.882	—	—
43	79-09-4	Propionic acid	Sour	MS/RI/O	—	—	0.474 ± 0.041
44	503-74-2	Isovaleric acid	Rancid	MS/RI/O	3.454 ± 0.703	2.061 ± 0.508	2.681 ± 0.014
45	105-43-1	dl-3-Methylvaleric acid	Rancid	MS/RI/O	—	28.676 ± 3.099	11.064 ± 2.156
46	150-86-7	Phytol	Green	MS/RI/O	—	17.656 ± 1.18	11.731 ± 1.047
47	123-35-3	Myrcene	Wood, pines	MS/RI/O	9.744 ± 0.822	—	—
48	138-86-3	dl-Limonene	Pines, fruity	MS/RI/O	443.202 ± 18.155	58.308 ± 0.416	86.796 ± 20.674
49	673-84-7	2,6-Dimethyl-2,4,6-octatriene	Sweet	MS/RI/O	9.099 ± 0.546	—	—
50	504-96-1	7,11,15-trimethyl-3-methylidene-hexadec-1-ene	Green	MS/RI/O	368.02 ± 11.445	237.276 ± 6.621	168.967 ± 1.119
51	60-35-5	Acetamide	Musty	MS/RI/O	—	2.782 ± 0.945	—

The highest content of aroma compounds was found in YXYQ with 1582.52 μg/L, followed by DHYQ with 916.736 μg/L, and the lowest content of aroma compounds was found in PEYQ with 656.676 μg/L. This result coincided with the high content of aroma compounds in the tobacco. In the three types of tobacco, the top two most abundant compounds were the same, terpenoids and heterocyclic compounds, which were the highest in YXYQ, followed by DHYQ, and lastly PEYQ, and the contents varied greatly. Among them, terpenoids were the most abundant, reaching 830.065, 295.584, and 255.763 μg/L in YXYQ, DHYQ, and PEYQ, respectively, with a high percentage of 52% in YXYQ, followed by heterocyclic compounds, with contents of 346.126, 272.151, and 196.297 μg/L. In addition, the contents of the compounds (237.73 μg/L) and ketones (141.055 μg/L) in YXYQ, aromatic compounds (215.089 μg/L) and ketones (82.737 μg/L) in DHYQ, and ketones (93.885 μg/L) and aromatic compounds in PEYQ. The compounds that were more abundant in PEYQ were ketones (93.885 μg/L) and aromatic compounds (84.781 μg/L).

### 3.5 Comparative analysis of tobacco and smoke aroma compounds

**FIGURE 8:**
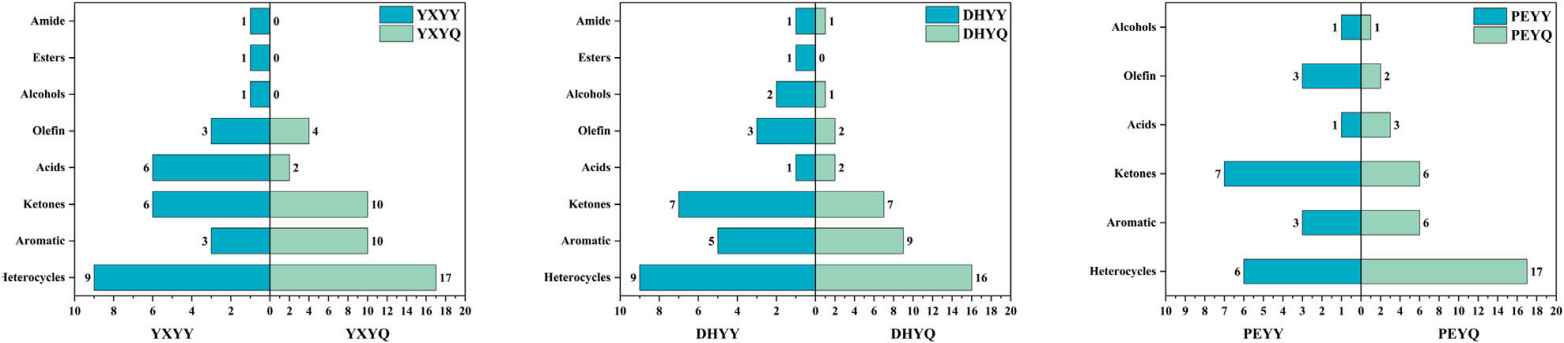
Comparison of tobacco and tobacco aroma compound types.

In order to visualize the comparison of aroma compounds in cigar tobacco and smoke, a comparison of species was plotted as shown in Figure 8. Compared to the aroma compounds in the smoke, there is a greater variety of aroma compounds in the tobacco, but the number of aroma compounds is much smaller than that in the smoke. During the combustion of YXYY to produce flue gas, amide (acetamide), ester (2-ethylhexyl acetate), and alcohol (phytol) compounds are decomposed in a series of reactions, resulting in a decrease in the number of aroma compounds in the flue gas. Among them, acetamide, as a nitrogen-containing organic substance, may undergo the following reactions under high temperature conditions: 1. Dehydration reaction: generates acetonitrile (CH_3_CN) and water (H_2_O); 2. Decarboxylation reaction: decomposes into methane (CH_4_), hydrogen cyanide (HCN), and carbon monoxide (CO); 3. Oxidation reaction: generates carbon dioxide (CO_2_), ammonia (NH_3_), and water (H_2_O); 4. Intermediate products: May produce nitrogen-containing heterocyclic compounds (e.g., pyridine, pyrrole derivatives) or amines (e.g., methylamine) (Zhang et al., 2024). This is confirmed by the detection of seven new nitrile compounds in the volatile compounds of the flue gas. 2-Ethylhexyl acetate belongs to the group of ester compounds whose molecular structure contains acetyl and 2-ethylhexyl groups, which may decompose or oxidize during combustion or pyrolysis to form a variety of small-molecule organic compounds and gas (Wang et al., 2021). When plant alcohols are burned or pyrolyzed at high temperatures, the hydroxyl group (-OH) and the long-chain hydrocarbon group in their molecular structure may be broken and olefinic compounds (e.g. isoprene, limonene, etc.) may be produced (Zheng et al., 2022). In more detail, during the combustion process, lipids present in tobacco (e.g. undergo β-oxidative cleavage to generate low-molecular-weight alkenes (e.g. ethylene and propylene). Concurrently, alcohols such as phytonols undergo dehydration reactions to produce terpene compounds like isoprene. These olefinic substances not only increase the overall content of olefinic aromatic components in flue gas by approximately 40%–60%, but also contribute to the perception of fresh, grassy, and fruity aroma notes. Studies have demonstrated that the pyrolysis efficiency of certain unsaturated fatty acids can exceed 75%, indicating their significant role as precursor compounds for the characteristic aroma of flue gas (McGrath et al., 2003). These substances are important components of the burning aroma of tobacco, ketones (e.g., methylcyclopentenone, 2,5-dimethylcyclopentanone, etc.), which are compounds with distinctive aroma characteristics, are commonly found in the pyrolysis products of cigars (Banožić et al., 2020).

In the process of YXYY after combustion to generate smoke, the number of heterocyclic, aromatic, ketone and olefin compounds increased, in which the heterocyclic compounds from 9 kinds of tobacco after combustion to 17 kinds of smoke, mainly due to the pyridine, pyrazine compounds generated in large quantities, pyrazine and pyridine compounds as a nitrogen heterocyclic compounds, is the formation of the characteristic aroma of cigar tobacco key aroma components, so that the tobacco has a rich, full-bodied baking aroma and other pleasing aroma, significantly enhance the complexity of the aroma level. Tobacco has a rich and full-bodied baking aroma, bread aroma and other pleasant flavor, significantly enhance the complexity of the aroma level. The pyrazine and pyridine substances in the smoke, part of the original pyrazine and pyridine substances in the tobacco leaves from the direct transfer, part of the pyrazine substances from the pyrolysis process of amino acids and reducing sugars in the process of the Meladic reaction to generate new pyrazine aroma-causing substances (Xiang et al., 2021). The production of large amounts of pyrazines and pyridines during the combustion process of tobacco has been well studied (Wang et al., 2013). The increase in the number of aromatic compounds may originate from the pyrolysis of lignin and the secondary synthesis of polycyclic aromatic hydrocarbons (PAHs). Lignin is an important aromatic polymer in tobacco, and its pyrolysis generates monocyclic aromatic compounds, such as phenol, mainly through the fracture of the phenylpropane unit. At high temperatures, lignin is further demethylated and dehydroxylated to form polycyclic aromatic hydrocarbons (PAHs) (Ding et al., 2005). Ketones are mainly produced by oxidative cleavage of cellulose and hemicellulose. Cellulose undergoes a dehydration reaction at 200 °C-350 °C to produce levoglucan intermediates, which are further fractured to form small molecule ketones such as pyruvic aldehyde. The acetyl group of hemicellulose is directly removed in pyrolysis to generate ketones. The generation of olefins mainly involves dehydration and cleavage of lipids and sugars. Similar to YXYQ, the number of heterocyclic and aromatic compounds increased in DHYQ, while the number of alcohols and olefins decreased, and the rest of the compounds did not change much. The overall aroma compounds of PEYY and PEYQ were smaller than those of the first two samples, which coincided with the result that the overall sensory.

### 3.6 Source analysis of characteristic aroma substances

The smoke produced by cigar tobacco after combustion is the product of complex chemical reactions, and the formation of its aroma components involves the pyrolysis, oxidation and condensation of tobacco precursors. The cigar combustion process can be divided into three stages: low-temperature distillation (<250 °C): free volatile substances (such as nicotine, new phytodiene) through the distillation of the release, contributing to the initial aroma; medium-temperature pyrolysis (300 °C–600 °C): carbohydrates, amino acids, terpenes, and other precursors undergo pyrolysis to generate furan, ketones, aldehydes, and other neutral aroma components; high-temperature carbonization (>700 °C): protein and alkali cleavage to form nitrogen-containing heterocyclic compounds (such as pyridine, pyrazine), while producing harmful substances such as polycyclic aromatic hydrocarbons (McGrath et al., 2007). Aroma compounds in tobacco can be categorized according to precursors as ciphylline degradation products, carotenoid degradation products, phenylalanine conversion products, chlorophyll degradation products, and Melad reaction products.

**FIGURE 9:**
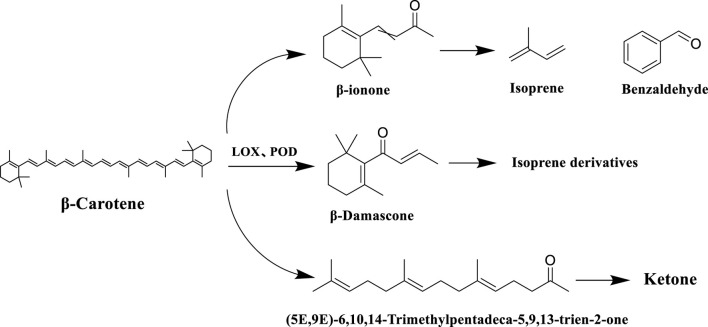
Degradation pathways of carotenoids in tobacco leaves.

Carotenoids is a general term for a group of important natural pigments, including β-carotene, lutein, etc., which are commonly found in plants and animals. Carotenoid degradation products, as the key neutral aroma component in the formation of the characteristic aroma of tobacco, its generation mainly involves a multi-stage transformation process in tobacco processing, including tobacco drying, fermentation, ripening and other stages of the process, and at present, more than 80 kinds of such compounds have been identified in tobacco, which have a very important impact on the aroma and aroma amount of the tobacco (Zhang et al., 2017). During tobacco fermentation, carotenoids are gradually degraded by enzymatic reactions under the action of enzymes (e.g. lipoxygenase LOX, peroxidase POD), generating congeners such as farnesyl acetone and megastigmene trienone, as well as small-molecule volatile compounds (e.g. β-violet ketone), which significantly enhance the aroma of tobacco, and have been detected in cigar tobacco in Yunnan province. When the tobacco is burned to produce smoke, the degradation products generated by fermentation (e.g. β-violet ketone) are further pyrolyzed to produce isoprene, benzaldehyde and other small molecule volatile compounds, which give the smoke a floral and fruity aroma and sweet flavor (Wang et al., 2020). The specific reaction processes of the carotenoid degradation pathway in tobacco are shown in Figure 9.

**FIGURE 10:**
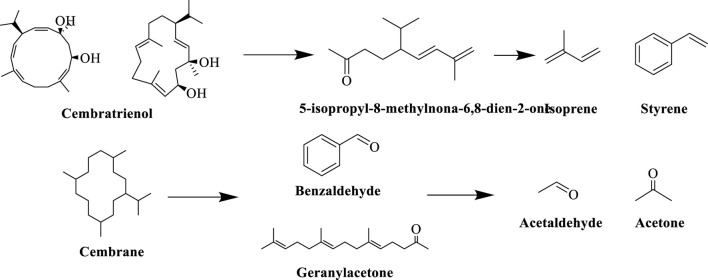
Degradation pathways of cedrans in tobacco leaves.

Cedrans are important diterpenoids in tobacco (Zhang, 2021), and lycopene, as its characteristic degradation product, is one of the most abundant neutral aroma-causing substances in tobacco, presenting tea and grassy aromas. During tobacco fermentation, microbial activities and enzymatic reactions dominate the degradation of cedrans, and dominant bacteria (e.g. *Staphylococcus* spp.) secrete hydrolytic enzymes through secondary metabolic pathways to break the cyclic structure of cedrans, generating small-molecule aldehydes and ketones, e.g., cedrans oxidized to produce benzaldehyde, geranylacetone, etc. (Song et al., 2023).When tobacco is burned (at temperatures >600 °C), cedrane degradation products undergo pyrolysis reactions: lycopene is pyrolyzed to isoprene, styrene and other small hydrocarbons; cedrane oxides are pyrolyzed to aldehydes (e.g. formaldehyde, acetaldehyde) and ketones (e.g. acetone) compounds. The specific reaction process of the degradation pathway of cedrans in tobacco is shown in Figure 10.

**FIGURE 11:**
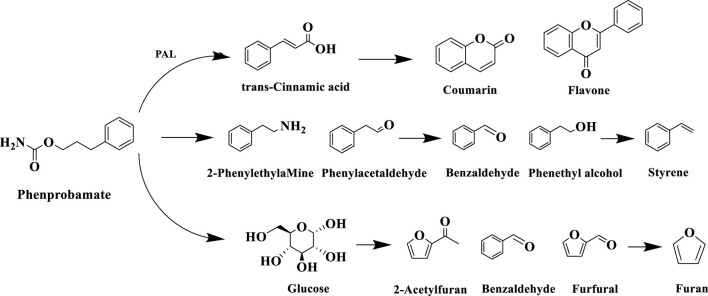
Comparison of tobacco and tobacco aroma compound types.

Phenylalanine (Phe), as an important precursor of tobacco aroma components, can be decomposed through its own reaction to produce benzaldehyde, benzyl alcohol, phenyl ethanol, phenyl acetaldehyde and other volatile small molecule compounds, and these degradation products contribute more to the fruity, clear aroma and other aroma-eating flavors of the tobacco, which have a direct impact on the formation of the aroma style of the tobacco. Gao et al. (2019) found that the degradation of phenylalanine in roasted tobacco during roasting is associated with phenylalanine deaminase (PAL), which is broken down into various aromatic compounds by the enzyme, which in turn produces small molecules such as phenylethanol. Phenylalanine, as a free aromatic amino acid, has been found to have three main metabolic pathways: one is deamidation, i.e., PAL-catalyzed generation of trans-cinnamic acid precursor, followed by biosynthesis of coumarins, lignin polymers, and flavonoids secondary metabolites through hydroxylation, methylation, and other modification reactions; the second is decarboxylation, i.e., under the action of the amino acid decarboxylase and peroxidase bi-enzymatic systems Under the action of amino acid decarboxylase and peroxidase dual enzyme system, it undergoes β-position decarboxylation to generate phenylethylamine and other amine intermediates; the third is the formation of pyrazines and furazones pyrolysis products through the Melad reaction with reducing sugar (Song et al., 2013). When tobacco is burned (>600 °C), the phenylalanine metabolites are further cleaved to produce small molecule compounds such as furan and styrene. The specific reaction process of the degradation pathway of phenylalanine in tobacco is shown in Figure 11.

**FIGURE 12:**
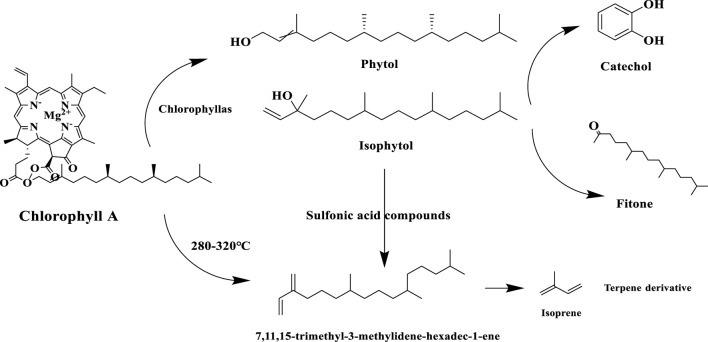
Degradation pathways of chlorophyll in tobacco leaves.

Chlorophyll degradation is an important biochemical reaction during tobacco maturation, conditioning and combustion, and its products, neophytadiene and phytol, play a key role in tobacco aroma quality. The results of the present study also showed that phytol contributed herbaceous and green aromas, which were the main contributors to the tobacco aroma characteristics of the three Yunnan cigar tobaccos. Chlorophyll is progressively degraded during tobacco maturation or fermentation via the demagnesyl chlorophyll a oxidase pathway (PAO pathway) (Ding et al., 2016), phytol production occurs in the first step: chlorophyll a is hydrolyzed by chlorophyllase to produce desmethyl chlorophyll a and phytol, followed by oxidation of phytol to produce phytocatechol, which is further involved in phytosterol metabolism (Gutbrod et al., 2021). Neophytadiene, on the other hand, is a dehydration product of phytol and is produced by enzymatic and non-enzymatic reactions. Phytol is catalyzed by dehydrating enzymes to produce neophytadiene during tobacco conditioning (e.g. roasting or fermentation). For example, isophytol is dehydrated in the presence of specific dehydration catalysts (e.g. sulfonic acid compounds) to produce neo- and trans-1,3-phytadiene (Gutbrod et al., 2021), or chlorophyll in the presence of glycerol, by 280 °C–320 °C high temperature cracking can be directly generated new plant diene. When the tobacco is burned, the new phytadiene undergoes a complex thermal cracking reaction to produce styrene and paste derivatives, which give the smoke sweet and woody aroma characteristics, and the phytol is decarboxylated at high temperatures to produce phytones and aldehydes (Rodríguez-Concepción Editor, 2025). The specific reaction processes of the chlorophyll degradation pathway in tobacco are shown in Figure 12.

**FIGURE 13:**
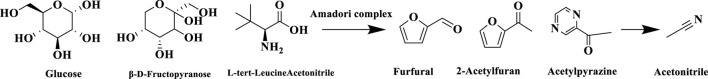
Maillard reaction in tobacco leave.

The Maillard reaction is a non-enzymatic reaction between reducing sugars and amino acids or proteins that occurs during heating or prolonged storage, resulting in the formation of volatile aldehydes, ketones and heterocyclic compounds. Maillard products in tobacco include furfural, furfuryl alcohol, 2-acetylpyrrole, 3-acetylpyridine, 2,3′-bipyridine and other aroma compounds (Zhou et al., 2024), it imparts roasted, burnt and smoky aroma to the tobacco. Glucose, fructose and other reducing sugars in tobacco condense with free amino acids to form Amadori complexes, which further degrade to form aldehydes and ketones, and heterocyclic compounds; when tobacco is burned, furfural compounds are cleaved to small aldehydes, and pyrazines to form nitrile compounds (Wang et al., 2016). The specific reaction process of the Maillard reaction in tobacco is shown in Figure 13.

## 4 Conclusion

This study conducted a systematic analysis of three Yunnan cigar tobacco varieties (YXYY, DHYY, PEYY), elucidating their characteristic chemical composition, which is marked by low sugar content (0.12%–0.16%), high nitrogen levels (2.84%–4.56%), and elevated chloride concentrations (1.16%–1.80%) that influence combustion properties. GC-MS analysis identified a total of 144 volatile compounds, among which heterocyclic (28.6%), aromatic (22.9%), and olefinic (18.1%) compounds were predominant, contributing significantly to the overall aroma profile. Notably, the core tobacco variety YXYY exhibited superior volatile complexity, comprising 120 identified compounds at a total concentration of 5131.226 μg/L, including key terpenoids (830.065 μg/L) and heterocyclic compounds (346.126 μg/L). During combustion, precursor compounds underwent transformations via Maillard reactions, resulting in a 32.7%–45.3% increase in key aromatic compounds, as well as oxidative degradation pathways, which led to a 12.8%–19.4% decrease in esters and alcohols. Carotenoid derivatives, particularly β-ionone, played a crucial role in shaping the final aroma characteristics. These findings establish a chemical foundation for enhancing tobacco quality through optimized agronomic practices, such as potassium management, and improved processing techniques, including fermentation control. Future research should focus on elucidating chloride metabolism and optimizing pyrolysis pathways to further enhance aroma development.

In addition, the research results have reference value for similar climate zones (such as Sichuan and Hubei in China), but adjustments need to be made in combination with regional differences. Compared with Cuban tobacco leaves, Yunnan tobacco leaves show characteristics of high nitrogen, high alkaloid and ion ratio imbalance, suggesting that it is necessary to optimize planting (such as potassium fertilizer regulation) and processing techniques (such as fermentation control) based on local conditions. In the future, efforts should be focused on optimizing the chlorine metabolism mechanism and pyrolysis pathways, and regional quality improvement strategies should be formulated through cross-region comparative studies.

## Data Availability

The original contributions presented in the study are included in the article/supplementary material, further inquiries can be directed to the corresponding authors.
